# Present Status, Challenges, and Prospects of Dihydromyricetin in the Battle against Cancer

**DOI:** 10.3390/cancers14143487

**Published:** 2022-07-18

**Authors:** Jiajun Wu, Zuowei Xiao, Hongfang Li, Neng Zhu, Jia Gu, Wenmao Wang, Chao Liu, Wei Wang, Li Qin

**Affiliations:** 1 Laboratory of Stem Cell Regulation with Chinese Medicine and Its Application, School of Pharmacy, Hunan University of Chinese Medicine, Changsha 410208, China; wujiajun10541@163.com (J.W.); 003831@hnucm.edu.cn (Z.X.); l15985025887@163.com (H.L.); gu583767721@163.com (J.G.); 2 Department of Urology, The First Hospital of Hunan University of Chinese Medicine, Changsha 410208, China; zhuneng10541@163.com; 3 Zhangjiajie Meicha Technology Research Center, Hunan Qiankun Biotechnology Co., Ltd., Zhangjiajie 427000, China; 13907442657@163.com (W.W.); liuchao3333@icloud.com (C.L.); 4 TCM and Ethnomedicine Innovation & Development International Laboratory, School of Pharmacy, Hunan University of Chinese Medicine, Changsha 410208, China; wangwei402@hotmail.com; 5 Institutional Key Laboratory of Vascular Biology and Translational Medicine in Hunan Province, Hunan University of Chinese Medicine, Changsha 410208, China; 6 Hunan Engineering Technology Research Center for Bioactive Substance Discovery of Chinese Medicine, Hunan University of Chinese Medicine, Changsha 410208, China

**Keywords:** dihydromyricetin, anticancer activity, multidrug resistance

## Abstract

**Simple Summary:**

With the advancements in diagnosis and treatment technology, the mortality of patients with cancer is gradually decreasing. However, further curbing cancer development requires searching for innovative and more effective drugs. Natural herbal medicine has apparent advantages in R&D and healthcare costs and is attracting increasing attention. Dihydromyricetin is a flavonoid compound with a promising anticancer effect extracted from the stems and leaves of *Ampelopsis grossedentata*. This paper reviews the different mechanisms of dihydromyricetin alone or in combination with other drugs against a variety of cancers to provide a comprehensive reference for the development of dihydromyricetin as an anticancer therapeutic agent.

**Abstract:**

Dihydromyricetin (DHM) is a natural flavonoid compound extracted from *Ampelopsis grossedentata* that has been used for centuries in traditional Chinese medicine. DHM has attracted intensive attention due to its numerous beneficial activities, such as hepatoprotection, cardioprotection, antioxidant, and anti-inflammation. In addition, DHM inhibits the progression of cancers such as lung cancer, hepatocellular cancer, breast cancer, melanoma, and malignant reproductive systems through multiple mechanisms, including antiangiogenesis, antiproliferation, apoptosis, and inhibition of invasion and migration. Notably, DHM also activates autophagy at different levels, exerting a dual-regulatory effect on cancers. Mechanistically, DHM can effectively regulate mammalian target of rapamycin (mTOR), noncoding RNA-mediated signaling, phosphatidylinositol 3-kinase (PI3K)/protein kinase B (Akt) pathway, nuclear factor-κB (NF-κB), p53, and endoplasmic reticulum stress (ER stress)-driven signaling in different types of cancers. DHM has also been shown to have inhibitory effects on various regulators that trigger epithelial–mesenchymal transition (EMT). Furthermore, DHM exhibits a remarkable anticancer reversal ability when used in combination with drugs such as adriamycin, nedaplatin, and other drugs. However, the low bioavailability of DHM limits its potential applications, which are improved through structural modification and the exploration of novel dosage forms. Therefore, DHM may become a promising candidate for treating malignancies alone or combined with conventional anticancer strategies used in clinical practice.

## 1. Introduction

Dihydromyricetin (3,5,7,3′,4′,5′-hexahydroxy-2,3-dihydroflavonol), known as ampelopsin, is a natural dihydroflavonol compound that was initially isolated from the stems and leaves of *Ampelopsis meliaefolia* by Kotake and Kubota in 1940 [1]. They found that the content of DHM in *Ampelopsis grossedentata* was the highest, reaching more than 30% [2]. Moreover, DHM is also widely found in Myrica, Cuculidac, Clusiaceae, Euphorbiaceae, and other plants [3]. *Ampelopsis grossedentata* has long been utilized in traditional Chinese medicine. In the past, the Yao minority of Guangxi, Hunan, in China, made vine tea from its stems and leaves to alleviate cold and fever, sore throat, jaundice, hepatitis, and other symptoms, as well as prevent and treat nephritis, halitosis, and polyphagia [4].

DHM has multiple pharmacological effects, including anti-inflammatory, antioxidant, and hepatoprotective properties. At present, DHM prescription is recommended for treating various diseases, such as alcohol use disorders and metabolic or neurological imbalances [5]. DHM capsules have been marketed in the United States as a nutritional supplement to prevent alcohol hangovers [6]. Modern pharmacological studies have shown that DHM exhibits a significant inhibitory effect on liver cancer [7], lung cancer [8], breast cancer [9], and so on. Furthermore, DHM has an excellent synergistic effect combined with other anticancer drugs, with low cytotoxicity or adverse effects on normal cells or tissues. Acute/chronic toxicity experiments and genotoxicity experiments of DHM have shown that the toxicity of DHM is very weak. For example, the maximum tolerance in rats administered with DHM by gavage is 5.0 g/kg body weight [6]. These beneficial properties have made DHM a hotspot in the research and development of anticancer drugs in recent years.

## 2. Antioxidant and Anti-Inflammatory Activities of DHM

Cell metabolism produces reactive oxygen species (ROS), which act as intracellular signaling molecules [10], and a relative excess of ROS can cause oxidative stress [11]. Once antioxidant defenses are insufficient against oxidative stress, ROS may contribute to various diseases [12]. Thus, targeting antioxidant capacity may have beneficial therapeutic effects. A variety of polyphenolic compounds have been shown to possess potent antioxidant properties, enhancing the expressions of antioxidant enzymes through the nuclear factor erythroid 2-related factor 2 (Nrf2)-mediated pathways [13]. As a redox-sensitive transcription factor, Nrf2 plays a critical role in maintaining redox homeostasis by inducing the expression of antioxidant-related NADH quinone dehydrogenase 1 (NQO1), heme oxygenase-1 (HO-1), and other factors [14]. DHM can activate the Nrf2/Kelch-like ECH-associated protein-1 (Keap1) pathway, sequentially increasing the expression of NQO1 in HepG2 cells in a dose- and time-dependent manner, and exert antioxidant activity [15]. It also has a strong scavenging ability of 1,1-diphenyl-2-picrylhydrazyl free radical (DPPH) and oxygen radical absorbance capacity (ORAC) in vitro [15]. DHM has also been reported to activate the Nrf2/HO-1 pathway, upregulate antioxidant enzymes and antiapoptotic proteins, and protect human umbilical vein endothelial cells (HUVECs) from oxidative damage induced by oxidized low-density lipoprotein (ox-LDL) [16]. In in vivo studies, DHM administration significantly reduced oxidative stress induced by sleep deprivation (SD) in the brains of Institute of Cancer Research (ICR) mice; superoxide dismutase (SOD) activity was improved, and malondialdehyde (MDA) levels were decreased in the hippocampus of SD mice [17]. In a cisplatin-induced mouse kidney injury model, DHM reduced MDA level in mouse kidney tissue by attenuating cisplatin-induced oxidative and inflammatory stress, increasing catalase (CAT) and SOD activities, thus protecting them from nephrotoxicity damage [18].

Chronic inflammation caused by the interaction of myeloid-cell-derived ROS with tumor necrosis factor-alpha (TNF-α)-mediated signaling may contribute to cancer progression [19]. DHM attenuated the inflammatory responses by inhibiting the NF-κB pathway, especially the activity of fibroblast-like synoviocytes (FLSs), in a rat model induced by interleukin-1β (IL-1β), thereby suppressing the proliferation, migration, and inflammatory responses of FLSs [20]. DHM can also inhibit doxorubicin-induced activation of the NLR family pyrin domain-containing 3 (NLRP3) inflammasome in H9C2 cells by blocking cysteinyl-aspartate-specific proteinase-1 (caspase-1) activity, suppressing IL-1β and IL-18 release, and upregulating sirtuin 1 (SIRT1) protein levels in vivo and in vitro [21]. In a mouse model of ovalbumin (OVA)-induced asthma, DHM reduced the number of inflammatory cells in bronchoalveolar lavage fluid and decreased the proinflammatory cytokine levels (such as IL-4, IL-5, IL-13) and secretion of ovalbumin-specific antibodies IgE and IgG1 in serum [22]. Furthermore, in a randomized, double-blind controlled clinical trial, DHM (150 mg capsules) twice daily for 12 weeks showed anti-inflammatory effects in patients with nonalcoholic fatty liver disease [23]. Oxidative stress and chronic inflammation are associated with the occurrence and development of diseases, especially cancers [24,25,26]. DHM has significant pharmacological effects in regulating inflammation and oxidative stress, which may be one of the reasons for the anticancer efficacy of DHM.

## 3. The Anticancer Potential and Underlying Mechanisms of Dihydromyricetin

### 3.1. Lung Cancer

According to the “latest Global Cancer Statistics 2020” released by the World Health Organization, lung cancer mortality has quadrupled over the past 40 years and has become the leading cause of cancer deaths in China (27.3%) [27]. Non-small-cell lung cancer (NSCLC) is a highly aggressive malignancy, accounting for more than 80% of total lung cancer patients. It is difficult to cure due to multidrug resistance and dose-dependent adverse reactions during the entire treatment process [28]. Studies have shown that DHM has anticancer activity against gefitinib-resistant and gefitinib-insensitive NSCLC cells, which causes apoptosis in NSCLC cell lines (A549 and H1975) by activating caspase-9/-7/-3 and cleaving poly ADP-ribose polymerase (PARP) [29]. However, it does not affect normal lung (WI-38) fibroblasts [29], indicating that DHM has fewer toxic side effects in anticancer therapy. Sustaining chronic proliferation during the cell cycle is the most fundamental trait of cancer cells [30]. Platelet-derived growth factors (PDGFs) have been shown to enhance the proliferation of fibroblast cells and extracellular matrix production [31], while interstitial fibroblasts can secrete various factors to increase the proliferative rate of cancer cells. It has been found that DHM can inhibit the PDGF-mediated proliferation of lung cancer fibroblasts by targeting the activation of the extracellular signal-regulated kinase (ERK1/2) and Akt pathways [32].

F-box proteins are an expanding family of eukaryotic proteins characterized by an “F-box” motif, which are responsible for substrate specificity in the ubiquitin–proteasome pathway, and play a pivotal role in programmed cell death [33]. F-box and WD repeat domain-containing 7 (FBW7) is a well-studied F-box-containing protein that functions as a cancer suppressor by targeting the oncoprotein substrate c-Myc for ubiquitination and degradation, thus regulating cell proliferation, apoptosis, and metabolism [34]. During DHM-induced apoptosis in A549 cells, the level of c-Myc protein levels and the expression of FBW7 were reduced in A549 cells [35], indicating that the ability of DHM to induce apoptosis is independent of FBW7. It is suggested that DHM can be used in drug-resistant cancers related to FBW7 deficiency.

### 3.2. Breast Cancer

Epidemiological studies have shown that the incidence of breast cancer increases rapidly during the reproductive years and gradually decreases around the age of 50 [36]. It has been proposed that treatment of breast cancer cell lines MCF-7 and MDA-MB-231 with DHM for 24 h results in a dose-dependent inhibition of cell viability in the above cell lines, but there is no toxic effect on human normal breast epithelial MCF-10A cells [37]. DHM can inhibit mitochondrial oxidative phosphorylation (OXPHOS) and reduce adenosine triphosphate (ATP) production, leading to mitochondrial damage and causing accumulation of ROS, increased mitochondrial membrane permeability, and activation of Bcl-2-associated X protein (Bax), ultimately inducing apoptosis [38]. In addition, DHM-stimulated ROS accumulation also leads to an ER stress response, which in turn upregulates GRP78, p-PERK, and CHOP expression, thereby promoting apoptosis of breast cancer cells. Conversely, severe ER stress in turn promotes the production of ROS [37]. These results show that DHM-induced ROS generation and ER stress in breast cancer may generate a vicious loop that increases its inhibitory effect on cancer cells. Accumulating evidence has also demonstrated that DHM is involved in regulating Akt/mTOR-pathway–mediated autophagy in addition to apoptosis. Autophagy is a lysosomal-mediated degradation process of intracellular senescent proteins or damaged organelles [39]. Generally, upon autophagy affection, tumor cells show strong viability in harsh extracellular environments such as metabolic stress, hypoxia, nutrient deficiency, and anticancer therapy [40,41]. Experimental studies have shown that DHM promotes the conversion of microtubule-associated protein light-chain 3B-I (LC3B-I) to autophagosome-associated LC3B-II, downregulates the expression of p62, and induces autophagy to protect breast cancer cells from apoptosis through the ER-stress-mediated Akt/mTOR pathway [42]. These findings may contribute to the rationale for enhancing the efficacy of DHM against breast cancer by inhibiting protective autophagy.

### 3.3. Osteosarcoma

Osteosarcoma mainly occurs in the long bones of adolescents and children, including the humerus, ulna, radius, femur, fibula, and pelvis, and is the most common primary malignant bone cancer [43]. DHM protects osteosarcoma MG63 cells against oxidative-stress-induced apoptosis by downregulating caspase activation and upregulating B-cell lymphoma-2 (Bcl-2) [44]. It is known that the urokinase plasminogen activator (uPA) is a serine proteinase that increases the amount of plasmin generated by plasminogen, thereby disrupting the extracellular matrix (ECM) and promoting cancer invasion and migration [45]. DHM has been found to reduce the expression of transcriptional factorS SP-1 and NF-κB from the cytoplasm to the nucleus via the ERK pathway, thereby reducing the ability of SP-1 and NF-κB to bind to the downstream uPA promoter, resulting in downregulation of uPA expression, and ultimately suppressing osteosarcoma metastasis [46]. Matrix metalloproteinases (MMPs) are members of a family of zinc-dependent proteolytic enzymes. The overexpression of MMP-2 and MMP-9 is associated with inflammation, tissue repair, and cancer progression metastasis [47]. DHM also blocks the migration and invasion of U2OS osteosarcoma cells by inhibiting MMP-2 expression stimulated by TNF-α [48]. Meanwhile, the G2/M-phase ratio is dramatically increased, which is related to the inactivation of GSK-3β in osteosarcoma cells with DHM treatment by stimulating 5′adenosine monophosphate-activated protein kinase α (AMPKα), resulting in upregulation of p21 expression and downregulation of Sox2 expression, thereby arresting the cell cycle [49].

### 3.4. Reproductive System Cancer

Ovarian cancer has one of the highest mortality rates among gynecological malignancies, with a high metastasis [50]. Notably, DHM can effectively inhibit the proliferation of ovarian cancer cells and induce cell apoptosis without apparent cytotoxicity to human ovarian surface epithelial cells [51]. EMT usually plays an important role in cancer progression, which refers to the fact that cells lose epithelial characteristics and acquire mesenchymal properties under special physiological or pathological conditions [52]. During ovarian cancer development, the occurrence of EMT can induce MMP production and accelerate the dissemination of cancer cells, thereby leading to the poor prognosis of ovarian cancer patients [53]. Moreover, DHM can block EMT via the NF-κB/Snail signaling pathway, upregulate E-cadherin expression, and downregulate the expressions of N-cadherin and vimentin in a concentration- and time-dependent manner, thus reducing the migration of ovarian cancer [54]. DHM also impairs the motility and invasiveness of ovarian cancer SKOV3 and A2780 cells and stimulates the c-Jun N-terminal kinase (JNK)/ERK-caspase-3 pathway, leading to apoptosis by upregulating the cleaved caspase-3 and Bax/Bcl-2 ratio in ovarian cancer cells [55].

Choriocarcinoma is another malignant gynecological tumor of uterine origin that usually occurs after a hydatidiform mole, abortion, or full-term delivery. Since it is derived from placental trophoblasts, it is also called gestational trophoblastic cancer. Choriocarcinoma is currently considered the most aggressive type of trophoblastic tumor [56] and is particularly prone to early distant metastasis, resulting in a poor prognosis [57]. Studies have found that DHM can cause S/G2/M cycle arrest in human fetally derived trophoblast choriocarcinoma JAR cells by downregulating the expressions of cyclin A1, cyclin D1, Smad family member 3 (Smad3), and Smad4 [58]. In addition, DHM can also induce JAR cell apoptosis by increasing the Bax/Bcl-2 ratio and decreasing the expression of pro-caspase-3 [59].

Prostate cancer is the most common urinary system malignancy in men. DHM effectively suppresses the proliferation of prostate cancer cells by reducing angiogenesis and prevents in vitro migration and invasion of PC-3 human prostate cancer cell lines by downregulating the expression of C-X-C motif chemokine receptor 4 (CXCR4) [60]. However, the inhibitory effect of DHM on the proliferation of normal prostate epithelial cells is much lower than that of prostate cancer cell lines [60]. Taken together, DHM affects the progression of reproductive system malignancies by preventing EMT, delaying angiogenesis, triggering apoptosis, and arresting the cell cycle.

### 3.5. Hepatocellular Carcinoma

Hepatocellular carcinoma (HCC) accounts for more than 90% of primary liver cancer and is the most common pathological type. DHM can reduce Akt expression and increase p53 levels via the Akt/Bad signaling pathway, which in turn increases caspase-3 cleavage and its downstream target PARP, resulting in apoptosis of HepG2 cells [61,62]. In particular, DHM stimulates the p53 protein, thereby inhibiting the degradation of mouse double minute 2 homolog (MDM2), a negative regulator of p53 [63], and promoting the expression of the death receptors death receptor-4 (DR4) and death receptor-5 (DR5), which induces apoptosis in liver cancer hepatoma cells [64]. Notch1 is a multifunctional protein that performs a variety of key regulatory roles in cell differentiation, development, proliferation, and survival under variable conditions [65,66]. Its abnormal expression usually causes cancer. DHM can downregulate Notch1 expression and decrease the Bcl-2/Bax ratio in hepatoma cell lines QGY7701 and HepG2 in a time- and dose-dependent manner, inhibiting the proliferation of hepatoma cells [67]. DHM downregulates the expression of MMP-9 protein by increasing the level of protein kinase C-δ (PKC-δ) protein and inhibiting the phosphorylation of p38, ERK1/2, and JNK, which significantly suppresses the migration and invasion of human hepatoma SK-Hep-1 and MHCC97L cells [68].

Nicotinamide adenine dinucleotide phosphate oxidase 4 (NOX4) is a key enzyme involved in ROS production [69]. It has been illustrated that DHM can downregulate the expressions of transforming growth factor β (TGF-β) and NOX4 in mouse hepatoma Hepal-6 cells, thereby reducing the production of ROS and ATP, inducing cancer cell apoptosis and avoiding damage to normal cells [70]. Although multiple pathways regulate the autophagy process in vivo, the mTOR signaling pathway is the most important. DHM induces autophagy by blocking the activation of mTOR at the stage of autophagosomes and acidic lysosomes, then impeding the proliferation of HepG2 cells [71].

### 3.6. Gastric Cancer and Cholangiocarcinoma

Gastric cancer is a malignant tumor originating from the gastric mucosa. Due to the lack of specificity and significance of early symptoms, more than 80% of gastric cancer patients in China are already in the advanced stage at the time of diagnosis, and combination therapy with fluorouracil and platinum is the first-line treatment for advanced gastric cancer [72]. However, its adverse effects are more prominent, and primary resistance or acquired resistance can ultimately lead to treatment failure and poor prognosis in patients with gastric cancer [73]. It has been found that DHM exerts anticancer effects by increasing the expression of p53 mRNA in a dose- and time-dependent manner and inhibiting Bcl-2 mRNA coding, as well as activating the endogenous mitochondrial pathway to induce apoptosis of AGS human gastric cancer cells [74]. DHM was also found to downregulate MMP-2 expression and inhibit the migration of human gastric cancer MKN45 cells through the JNK pathway [75]. High-motility group box 1 (HMGB1) plays a critical role in the enhancement of tumor angiogenesis and suppression of host anticancer immunity [76]. A study has shown that DHM reduces HMGB1 expression and suppresses the Akt/STAT3 pathways, thereby inhibiting the proliferation and migration of gastric cancer cells [77].

MicroRNAs (miRNAs) are small endogenous noncoding RNAs that play dual roles in cancer progression according to their targets. For example, microRNAs (miRNAs) located in amplified regions of the cancer genome (such as the miR-17-92 cluster) function as oncogenes, while miRNAs located in the deleted part of the cancer chromosome (such as the miR-15a-miR-16-1 cluster) have cancer-suppressing gene function [78,79]. Among them, miR-455 is a suppressor in various gastrointestinal cancers such as colorectal and pancreatic cancer [80,81], while miR-21 is an antiapoptotic and prosurvival factor [78]. DHM can enhance miR-455 expression, which decreases the expression of ZEB1, p-PI3K, and p-Akt in cholangiocarcinoma cell lines RBE and TFK-1 and suppresses cell proliferation and EMT in cholangiocarcinoma [82]. In addition, DHM also reduces the expression of miR-21, phosphorylated Akt, and MMP-9 and upregulates the expression of phosphatase and tensin homolog (PTEN) via the miR-21/PTEN/Akt pathway, attenuating the proliferation and invasion of human cholangiocarcinoma cells [83].

### 3.7. Colorectal Cancer

Colorectal cancer is a common malignant tumor of the digestive tract. Its early symptoms are usually not obvious, and 20–25% develop distant metastases at diagnosis [84]. The primary option for colorectal cancer treatment to prolong the survival of patients is chemotherapy intervention [85]. However, the efficacy of currently approved therapeutic drugs is quite limited, which can only increase survival for several months [86].

DHM can activate phosphorylation of AMPK and upregulate XIAP-associated factor 1 (XAF1) through ROS-mediated ER stress; subsequently, it upregulates XAF1 and disrupts mitochondrial membrane potential, leading to apoptotic death in colon cancer [87].

Studies have shown that inflammatory bowel disease is a significant risk factor for colon cancer [88,89,90]. DHM may fight against colon cancer by intervening in colitis-related factors. Butyrate, an important fiber metabolite in the gut, protects the host from colitis-associated colon cancer by enriching the genus prevotella. DHM has been found to delay colon cancer formation by maintaining lower butyrate levels during the hyperproliferative phase and by acting as an activator of chloride channels (CFTR, CLCN3, and CLCN4); 100 mg/kg DHM can also improve the dominant intestinal flora in high-fat-diet–fed cancer mice and reduce the susceptibility to colon cancers [91]. Human semaphorin 4D (Sema 4D) is highly expressed in a variety of aggressive cancers [92] and serves as a compensatory angiogenic factor to potentiate cancer growth and angiogenesis [93]. DHM has been demonstrated to significantly lower Sema4D expression in vivo and in vitro, resulting in a reduction in inflammatory invasion and limiting the expression of inflammatory factors and MDA levels in CoLo-205 colorectal cancer cells [94], reducing the risk of cancer development.

### 3.8. Melanoma

Malignant melanoma is the most aggressive and treatment-resistant form of skin cancer. Its aggressiveness is based on the highly metastatic potential of melanoma cells, even in the early stages [95]. DHM can affect melanoma initiation, invasion, and migration in multiple ways. DHM downregulates the expression of Cdc25A, Cdc2, and P-Cdc2, blocking the cell cycle progression, resulting in a decrease in S-phase and G2/M-phase cells and a substantial increase in the proportion of G1-phase cells in SK-MEL-28 cells [96]. A previous study has also found that DHM induces apoptosis of SK-MEL-28 cells by increasing the Bax/Bcl-2 protein ratio and caspase-3 activity in a dose- and time-dependent manner [97]. Furthermore, DHM also rapidly activates autophagy through ROS-NF-κB signaling, which provides a cytoprotective mechanism for human melanoma cells and attenuates DHM-induced apoptosis [97]. In in vivo experiments, DHM can suppress the formation of lung metastases by blocking the invasion of B16 cells across the vascular subendothelial basement membrane, thereby inhibiting cell invasion and metastasis in a mouse model of B16 melanoma artificial lung metastasis [98]. In summary, inhibition of invasion and metastasis is the key step in melanoma therapy. However, the molecular targets of DHM are not yet precise and need further exploration.

### 3.9. Squamous Cell Carcinoma

**Figure 1:**
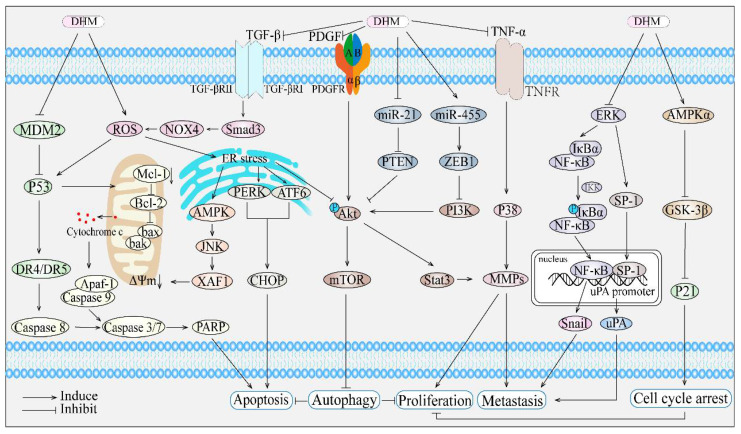
Anticancer mechanisms of dihydromyricetin. DHM regulates MDM2-mediated p53 pathway to trigger apoptosis in exogenous death receptor pathway and inhibits TGF-β-Smad3 signaling, disturbs ROS balance, and promotes apoptosis in endogenous mitochondrial and endoplasmic reticulum pathways. DHM also induces autophagy through the PDGFR/Akt/mTOR pathway, inhibiting cell proliferation. In addition, DHM inhibits miR-21/PTEN/Akt pathway, miR-455/ZEB1/PI3K pathway, TNF-α/P38/MMP pathway, and ERK/NF-κB/Snail pathway, as well as ERK/SP-1/uPA pathway, thereby suppressing cancer cell metastasis and proliferation. Furthermore, DHM can also activate AMPK α/GSK-3β/P21 pathway, leading to cell cycle arrest.

Head and neck squamous cell carcinoma (HNSCC) is an aggressive, life-threatening disease that constitutes 90% of head and neck carcinomas (HNCs) [99], and DHM has good pharmacological activity against HNSCC. It has been demonstrated that DHM enhances p-STAT3-dependent autophagy by activating the ROS-signaling pathways, which provides a cytoprotective mechanism in HNSCC cells [100]. Metastasis-associated lung adenocarcinoma transcript 1 (MALAT1) is one of the highly conserved long noncoding RNAs, and its expression is closely related to the progression of malignant cutaneous cancer [101,102]. In cutaneous squamous cell carcinoma, DHM can inhibit the phosphorylation of malat1-transcription factor EB (TFEB), thereby activating TFEB nuclear translocation, increasing the activity of the TFEB reporter gene and the expression of autophagy-related genes and decreasing the expression of MALAT1, thereby disturbing MALAT1 homeostasis and promoting autophagic cell death in A431 cells [103]. DHM also has a certain anticancer effect on another common HNSCC, nasopharyngeal carcinoma (NPC). It is a nonlymphomatous squamous cell carcinoma derived from epithelial cells lining the nasopharynx [104]. DHM can silence the activation of p-IKKβ/α, block the nuclear translocation of NF-κB subunit p65, significantly suppress TNF-α-mediated NF-κB activation in CNE-2 cells, and reduce the expression levels of Bcl-2 and pro-caspase-3, resulting in CNE-2 cell apoptosis [105]. Moreover, DHM can inhibit the migration and invasion of NPC cells by suppressing the expression of MMP-2 by downregulating the ERK1/2 signaling pathway [106]. Figure 1 shows the anticancer mechanism of DHM.

## 4. Synergistic Effects of Dihydromyricetin with Anticancer Agents

**Figure 2:**
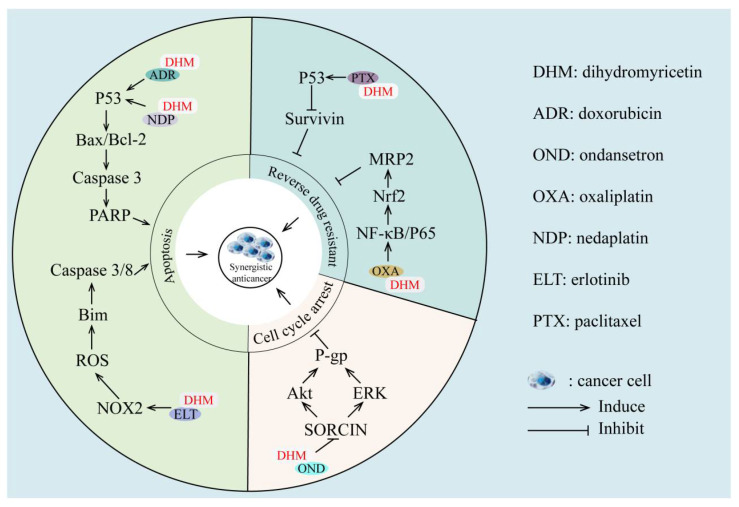
Synergistic effects and mechanisms of DHM combined with different chemotherapeutic drugs. DHM is used in combination with a variety of drugs (such as ADR, OND, OXA, NDP, ELT, and PTX) to play a synergistic anticancer effect by increasing the sensitivity of cancer cells to drugs, reversing multidrug resistance, and inducing cancer cell death.

Chemotherapy is one of the traditional strategies of cancer therapy. However, chemotherapy usually fails due to the drug resistance of cancer cells, of which multidrug resistance (MDR) is the most common. It refers to the cross-resistance of cancer cells to chemotherapeutic drugs with multiple structures and modes of action. Previous studies have shown that the expressions of the MDR gene (MDR1, MDR2), P-glycoprotein (P-gp) [107], and the multidrug-resistance-associated protein gene (MRP) [108] are closely related to MDR. Several studies have demonstrated that combining DHM with chemotherapeutic medicines may enhance chemosensitivity, reversing MDR (Figure 2). Adriamycin (ADR), a commonly used anthracycline chemotherapy drug, can cause fatal congestive heart failure due to its severe cardiotoxicity [109]. DHM promotes the accumulation of the antiapoptosis protein ARC (apoptosis repressor with caspase recruitment domain), prevents cardiomyocyte apoptosis, and has a synergistic anticancer impact in vitro and in vivo. The above effects are mainly achieved by modulating MDM2-mediated degradation of ARC ubiquitination [110]. This indicates that DHM is expected to become an effective cardioprotective agent against toxicity and increase the therapeutic window of ADR. Moreover, the combination of DHM and ondansetron (OND) suppresses soluble resistance-related calcium-binding protein (SORCIN) and thus impairs the function of the transporter P-gp, promoting the arrest of the G2/M phase and apoptosis induced by ADR and elevating the antiproliferation effect of ADR [111]. Furthermore, DHM also attenuates the drug efflux mediated by P-gp and then increases the intracellular accumulation of chemotherapeutic drugs, thereby reversing the MDR of human leukemia K562/ADR cells [112]. In addition, DHM reverses the resistance to 5-FU in SGC7901/5-FU cells in a concentration-dependent manner by downregulating MDR1 mRNA expression and impeding the pumping effect of P-gp [113], indicating that DHM can be applied as an effective MDR reversal agent in the treatment of gastric cancer. It is known that the survivin gene is a member of the inhibitor of the apoptosis protein family, and the overexpression of survivin may provide growth and survival advantages for cancer initiation and progression [114]. DHM inhibits survivin expression and sensitizes ovarian cancer cells to paclitaxel and ADR dramatically, enhancing the effect of chemotherapy [51].

The combination of erlotinib (ELT) and DHM can increase the expression of Bcl-2-interacting mediator of cell death (Bim) and induce NOX2-ROS-dependent caspase apoptosis in erlotinib-resistant NSCLC cells through the NOX2-ROS-Bim pathway [115]. In addition, DHM also alters mitochondrial function by activating the p53/Bcl-2 pathway, promotes NDP-induced apoptosis of liver cancer cells, and reduces liver cancer cell damage by increasing the Bcl-2/Bax or Bcl-2/Bak ratio [116]. DHM also suppresses MRP2 transcription by blocking the Nrf2 pathway, reversing colorectal cancer cell resistance to oxaliplatin (OXA), and making colorectal cancer cells susceptible to OXA-induced death [117,118]. DHM promotes the anticancer effect of irinotecan (CPT-11) in colitis-related colon cancer and Min (APC min/+) mouse models [85]. The synergistic potential of DHM as an adjuvant to first-line anticancer drugs is gradually emerging. However, the low bioavailability of DHM still limits its activity. Therefore, DMH needs to be modified by medicinal chemistry to improve its efficacy in reversing drug resistance.

## 5. Challenges/Limitations

Despite the promising future of DHM in cancer therapy, its chemical instability and low bioavailability hinder its application. DHM is only soluble in hot water and ethanol and slightly soluble in water at 25 °C (200.3 μg/mL) [119]. For example, when rabbits were administered a dose of 115 mg/kg for 1.5 h, the plasma DHM concentration reached a maximum (159 μg/L), indicating a low bioavailability of DHM [120]. In addition, the phenolic hydroxyl structure of DHM makes it unstable. In particular, when DHM is exposed to light, pH buffers, pepsin, and trypsin, it will undergo various chemical reactions such as oxidation, hydrolysis, cleavage, and reduction, and decompose to produce metabolites [121]. Generally, DHM is stable in simulated gastric fluid and buffer solutions at pH 1.2 but undergoes pseudo-first-order kinetic degradation at pH 6.8 [122]. Pharmacokinetic studies have also shown that DHM is not easily absorbed into the blood and is unstable in the intestinal environment [123]. These results suggest that DHM may be metabolized and eliminated in the intestine. Therefore, gastrointestinal pH rather than digestive enzymes primarily affects the disintegration of DHM. DHM is slightly acidic due to the chemical structure of DHM containing many phenolic hydroxyl groups, which increases its stability under acidic conditions. Moreover, the transporters multidrug resistance protein 2 (MRP2) and breast cancer resistance protein (BCRP) inhibit DHM uptake and transport and block the absorption of DHM in the intestine [124].

DHM has regulatory effects on autophagy. It is known that autophagy can inhibit tumorigenesis by controlling the degradation of damaged components or proteins in cells [125]. Meanwhile, cancer cells can also utilize autophagy to adapt to various stresses, such as hypoxia or nutritional deficiency, to support the metabolism for tumor survival and excessive proliferation, and promote the growth of most advanced tumors [126]. There is currently a lack of adequate autophagy-based therapeutic interventions for cancer treatment. As previously described, DHM induces protective autophagy in breast cancer, melanoma, and HNSCC. On the contrary, DHM also prevents the occurrence and progression of hepatocellular carcinoma and cutaneous squamous cell carcinoma through autophagy. The stress-induced autophagy produced by DHM in different tumor cells exhibits multiple effects of anticancer therapy. Its specific effect may be related to different cancer types, stages of development, and genetic backgrounds [127]. Thus, an in-depth discussion of the unique roles of DHM-mediated autophagy in various types of malignancies in combination with tumor features is critical for rational selection of autophagy-targeted therapeutic options and eventual benefits.

Furthermore, a number of pathways have been revealed on how DHM regulates different cascades and inhibits/prevents cancer development. However, the precise targets of DHM signaling still need more exploration, and its pharmacological effects, distribution, and metabolic processes in animal models have not been comprehensively summarized. Evidence from clinical studies is also weak [23]. More in vivo studies through techniques such as multiomics are still required.

## 6. Strategies to Improve the Effects of DHM

### 6.1. Structural Modification

DHM has the disadvantages of low selectivity and weak pharmacological activity. Therefore, increasing the pharmacological activity and stability of DHM based on its structural characteristics and chemical modifications has potential prospects.

Generally, flavonoids can be acylated by using chemical or enzymatic methods. However, chemical acylation is not regioselective, which would lead to undesired functionalization of hydroxyl groups. In addition, enzymatic acylation is more regioselective and able to produce the desired acylation effect [128]. Previous studies have shown that regioselective enzymatic acylation of DHM with lipases from various sources can increase its solubility in lipid systems, with immobilized *Penicillium expansum* lipase and Novozyme 435 achieving the maximum conversion (85%) [129]. Furthermore, quantum chemical calculations have shown that the 3-OH of DHM has the lowest antioxidant activity, indicating that acylation has the least influence on antioxidant activity at this position [129]. Recently, Cao et al. used the novel deep eutectic solvent (DES)-DMSO cosolvent system as the reaction medium and utilized immobilized lipase from *Aspergillus niger* (ANL) to catalyze the enzymatic acylation of DHM. The results revealed that DHM-16-acetate (91.6% conversion) was 10 times more lipophilic than DHM and exhibited good antioxidant activity [130]. Notably, studies have also demonstrated that proper acylation can enhance the lipophilicity, antioxidant activity, and thermal stability of DHM. However, excessive acylation of DHM reduces antioxidant activity and thermal stability [131]. Therefore, moderate acylation helps DHM to exert its antioxidant activity.

Glycosylation can significantly affect drug solubility, stability, bioavailability, and antioxidant properties [132,133]. Jin et al. synthesized five kinds of ampelopsis glycosides (AMPLS Gs) by the reaction of glucan sucrase with ampelopsin and sucrose receptor. Among them, the water-solubility, free-radical-scavenging, and antibrowning abilities of AMPLS-G1 were markedly improved [134], showing the whitening and moisturizing function of DHM glycosides playing in cosmetics.

The molecular structure of DHM has super delocalizability; a complete, conjugated, large π bond; strongly coordinating oxygen atoms; and a suitable spatial configuration, making it a good metal-ion-chelating ligand [135]. Three potential domains for binding metal ions exist in the DHM structure, including the 3′,4′-dihydroxy group in the B ring and the 3-hydroxy or 5-hydroxy (in the A ring) and the 4-carbonyl groups in the C ring [136]. A previous study has demonstrated the interactions between bovine serum albumin (BSA) and DHM-Cu(II), DHM-Mn(II), and DHM-Zn(II) complexes. Different DHM-metal complexes can alter the transportation, disposition, and pharmacological effects of free DHM [137].

### 6.2. Drug Delivery Systems

Carrying traditional drugs through nanomaterials (micelles, liposomes, nanoparticles, etc.) can improve drug solubility, prolong drug half-life in vivo, and enhance bioavailability [138]. For example, DHM-loaded Solutol^®^HS15 micelles (DHM-Ms) prepared by the thin-film hydration method have better sustained-release properties than free DHM. In the single-pass intestinal perfusion model, the absorption rate constant (Ka) and permeability coefficient (Papp) of DHM-Ms are 5.5 and 3.0 times higher than those of pure DHM, respectively. The relative bioavailability of DHM-Ms (AUC_0–∞_) is 205% compared to free DHM (AUC_0–∞_), significantly increasing the bioavailability of DHM [139]. On the other hand, PEGylated dihydromyricetin-loaded liposome-encapsulated tea saponin grafted on chitosan (TS/CTS@DHM-Lips) as an effective cationic antibacterial agent, exhibiting good water solubility and aqueous solution stability capable of controlled release of DHM in weakly acidic and neutral physiological environments [140]. In addition, DHM-encapsulated zein caseinate nanoparticles (DZP) can be prepared by using zein nanoparticles as carriers to enhance the biostability of DHM. Data from UPLC-QqQ-MS/MS quantity analysis have demonstrated that the bioavailability of DHM in rats is increased by 1.95 times, and the concentration of its metabolites in rat plasma is also elevated, suggesting that the nanoparticles significantly improve the adhesion of DHM in the gastrointestinal tract of mice [141], which may facilitate the absorption of DHM by prolonging its residence time in the upper gastrointestinal tract [142].

Drug–drug cocrystals consisting of two or more active pharmaceutical ingredients (APIs) have been developed as a new class of solid-form APIs [143,144]. Studies have shown that the drug–drug (1:1) cocrystal hydrate of DHM and pentoxifylline (PTY) prepared by the slimy method achieves the simultaneous sustained release of DHM and PTY and has a synergistic anticancer effect on HepG2 cells in vitro at a concentration of 100 μM [145]. In addition, the DHM-loaded gastric-floating sustained-release tablet (DHM-GFT) is prepared by direct powder compression, and pharmacokinetic studies have shown that DHM-GFT has a good intragastric floating ability and sustained-release effect in an acidic environment, which could significantly prolong the retention time of drugs in the body and improve bioavailability [146].

## 7. Conclusions and Perspectives

Dihydromyricetin, a natural flavonoid with various beneficial biological activities, has significantly progressed in its role and mechanism in cancer prevention research (Table 1). DHM inhibits cancer development through antiangiogenesis, antiproliferation, apoptosis, and suppression of invasion and migration. In particular, DHM has a prominent effect in synergistic anticancer with traditional chemotherapeutic drugs, which can effectively reverse drug resistance and avoid severe adverse reactions. Although DHM has shown to have a therapeutic effect on certain cancers in in vivo and in vitro studies, its molecular mechanism remains unclear, particularly DHM-activated autophagy’s different roles in various cancers. Most studies on DHM focus on the cellular level, involving signaling pathways such as Akt/mTOR, NF-κB, and p53, and the target of direct antitumor function is still unknown. In particular, in clinical trials, no substantial progress has been made. A disadvantage of DHM is its relative lack of potency, requiring micromolar concentrations of the drug to be effective, limiting progress in in vivo and clinical studies. In addition, due to factors such as low solubility, DHM also suffers from poor bioavailability, which limits its pharmacological effects and clinical applications. To date, the bioavailability and activity of DHM have been enhanced by chemical modification and the development of novel drug delivery systems such as nanocapsules and self-assembled micelles. Taken together, DHM is a potential anticancer Chinese medicine monomer, which is worthy of in-depth study and development in the future.

## Figures and Tables

**Table 1:** Anticancer activities of dihydromyricetin.

Cancer Type	Cell Types (Animals)	Concentration of DHM	Upregulated Related Proteins	Downregulated Related Proteins	Effect of DHM	Ref
Lung cancer	A549, H1975	75 μM	Caspase-9/-7/-3; JNK1/2; ERK1/2	PARP; Bcl-w	Apoptosis	[29]
		30 μM		XIAP; survivin; HDAC2; c-Myc; Skp2; FBW7α; FBW7γ; GSK-3β	Apoptosis proliferation inhibition	[35]
		10 μM	ERK1/2; Akt		Proliferation inhibition	[32]
Hepatocellular carcinoma	HepG2	50 μM	Beclin-1; LC3-II; PI3K; AMPK	p-ERK1/2; p-Akt; PDK1	Invasion inhibition	[71]
		30 μM	Bax; caspase-3	Bcl-2	Apoptosis	[61]
		100 μg/mL	caspase-3/-9/-8; DR4; DR5; Bax; p53	Bcl-2	Apoptosis	[64]
	HepG2, Hep3B	200 μM	p-Chk1; p-Chk2; CDK1		Cycle arrest	[7]
	HepG2, QGY7701, Hepal-6	100 µM	p53; caspase-3	Bcl-2	Apoptosis	[62]
	QGY7701, HepG2	100 μM	Bax	Notch1; Hes1; Bcl 2	Apoptosis	[67]
	Hepal-6	100 μM		TGF-β; TGF-βRII; Smad; p-Smad2/3; NOX4; ROS; ATP	Apoptosis	[70]
	SK-Hep-1, MHCC97L	50–100 μM	PKC-δ	MMP-9; P-ERK1/2; JNK	Invasion inhibition	[68]
	HepG2, HL7702	50 μM	caspase-9/-8/-3; HO-1; BAK	ROS; GSH; ATP; Bcl-2	Apoptosis	[147]
Cholangiocarcinoma	HCCC9810, TFK-1	156.8 µM	Caspase-3; Bad; PTEN	p-Akt; Bcl-2; MMP9; vimentin; miR-21	Invasion inhibition	[83]
Colon cancer	Colo-205 (male Balb/c nude mice)	64 Μm (100 mg/kg)	GSH; CAT; SOD; GPX; HO-1	Sema4D; ROS; MDA; COX-2; iNOS	Proliferation inhibition	[94]
	HCT-116, HCT-8, HT-29	100 μM	GRP78; CHOP; p-AMPK; XAF1	p-p38; p-JNK; Bcl-2; Mcl-1	Apoptosis	[87]
Gastric cancer	AGS	25–100 μM	p53 mRNA	Bcl-2 mRNA	Apoptosis	[74]
	BGC-823	80 μg/mL		HMGB1	Proliferation inhibition	[77]
Breast cancer	MCF-7, MDA-MB-231	80 μM	ROS; GRP78; p-PERK; CHOP		Apoptosis	[37]
		60 μM	p-elF2α; cleaved ATF6α	p-Akt; p-mTOR; p-p70S6K	Autophagy	[42]
Human melanoma	SK-MEL-28	100 μM	Caspase-3; ROS; LC3; p62; Beclin-1		Apoptosis	[97]
		100 μM	p53; p21	Cdc25A; Cdc2; P-Cdc2	Cycle arrest	[96]
Human ovarian cancer	A2780	50 µM	E-cadherin; p65	N-cadherin; vimentin; Snail	Invasion inhibition	[54]
	SKOV3, A2780	120, 80 μM	caspase-3; Bax	Bcl-2; GRASP65	Apoptosis	[55]
Prostate cancer	LNCaP, PC-3 (male severe combined immune-deficient mice)	25 µM, 60 µM (300 mg/kg)		CDK2; Cdc2; Bcl-2; CXCR4	Invasion inhibition	[60]
Choriocarcinoma	JAR	100 mg/L		Smad3; p-Smad3; Smad4; cyclin A1; cyclinD1	Proliferation inhibition	[58]
		100 mg/L		caspase-3; Bax; Bcl-2	Apoptosis	[59]
Osteosarcoma cells	MG63	30 µM	Bcl-2	caspase-3/-9	Apoptosis	[44]
	U2OS, MG63, Saos2, HOS, 143B cells (athymic nude (nu/nu) mice)	60 μM (300 mg/kg)	p21; AMPKα; p38MAPK; GSK-3β; JNK	Sox2	Proliferation inhibition	[49]
	U-2OS, HOS	100 μM	IκBα	SP-1; NF-κB; uPA, ERK2	Invasion inhi-bition	[46]
	U2OS, MG63, HOS	60 μM	p21; AMPKα	GSK-3β; Sox2	Invasion inhi-bition	[48]
Nasopharyngeal carcinoma	CNE-2	160 μg/mL	p-IKKβ; p-IKKα	Bcl-2; pro-caspase-3	Apoptosis	[105]
	HONE-1, NPC-BM, NPC-39	100 μM		ERK1/2; MMP-2	Invasion inhibition	[106]
